# Efficacy and Safety of Yellow Light Monotherapy Versus Yellow Light Combined With 20% Vitamin C for the Treatment of Melasma: A Single‐Center, Randomized, Double‐Blind, Placebo‐Controlled Clinical Study

**DOI:** 10.1111/jocd.71146

**Published:** 2026-08-23

**Authors:** Dan Du, Xu Liu, Yuwei Huang, Shuwei Wu, Xian Jiang

**Affiliations:** ^1^ Department of Dermatology, West China Hospital Sichuan University Chengdu China; ^2^ Laboratory of Dermatology, Clinical Institute of Inflammation and Immunology, Frontiers Science Center for Disease‐Related Molecular Network, West China Hospital Sichuan University Chengdu China

**Keywords:** low‐level light therapy, melasma, vitamin C, yellow light phototherapy

## Abstract

**Background:**

Melasma treatment remains challenging due to limited effective and safe options. This study evaluated the efficacy and safety of yellow light phototherapy alone versus combined with 20% vitamin C in treating melasma.

**Methods:**

Seventy‐one patients were randomly assigned to receive twice‐weekly yellow light phototherapy combined with either 20% vitamin C solution (*n* = 35) or its corresponding vehicle (*n* = 36). The primary outcome was the change from baseline in modified Melasma Area and Severity Index (mMASI) score. Secondary outcomes consisted of changes relative to baseline in Physician Global Assessment (PGA), Subjective Satisfaction Assessment (SA), melanin index (MI), individual typology angle (ITA°), transepidermal water loss (TEWL), skin hydration content (SCH), skin pore and skin texture parameters.

**Results:**

At Week 12, the combination group exhibited significantly greater reductions in mMASI scores (−1.95 ± 2.11 vs. −0.69 ± 2.19, *p* = 0.027) and lower Physician Global Assessment (PGA) scores (4.48 ± 0.80 vs. 5.06 ± 0.54, *p* = 0.002) compared with the monotherapy group. The combination group exhibited a more remarkable decrease in melanin index (MI) (−13.07, 95% CI: −17.82, −8.32 vs. −3.16, 95% CI: −7.86, 1.54, *p* = 0.005) and a more prominent elevation in individual typology angle (ITA°) (6.23, 95% CI: 4.38, 8.08 vs. 1.23, 95% CI: −0.60, 3.05) (*p* < 0.001). Moreover, significant improvements in skin pore status and skin texture were noted in the combination group (*p* < 0.05). No statistically significant between‐group differences were found in transepidermal water loss (TEWL) and skin hydration (SCH). No adverse events occurred throughout the trial. The participant treatment satisfaction rate reached 60.00% in the combination group, which was significantly higher than 22.55% in the control group.

**Conclusion:**

Combined 20% vitamin C plus yellow light phototherapy yielded statistically superior melasma improvements relative to yellow light monotherapy, though the absolute reduction in mMASI was modest. The combination regimen preserved skin barrier function and exhibited good tolerability.

**Abbreviations**
AE: adverse event
ITA°: individual typology angle
LLLT: low‐level light therapy
MI: mean index of melanin
mMASI: modified melasma area and severity index
PGA: physician's global assessment
SCH: skin hydration
TEWL: transepidermal water loss
VAS: visual analog scale

## 1 Introduction

Melasma is a common acquired hyperpigmentation disorder characterized by symmetric, light‐to‐dark brown macules on the face with irregular borders, significantly impacting facial esthetics. The exact pathogenesis remains incompletely understood, though ultraviolet rays (UV) exposure is a well‐established exacerbating factor [1]. Additionally, genetic predisposition, pregnancy, thyroid disorders, oral contraceptives, and glucocorticoids are recognized risk factors for melasma development [2]. Although hydroquinone, retinoids, triple combination therapy, tranexamic acid, and laser therapies are widely recognized as effective treatments, high recurrence rates and adverse effects including post‐inflammatory hyperpigmentation persist [3].

Ascorbic acid, commonly known as vitamin C, demonstrates multifaceted efficacy in anti‐aging skincare. Acting as a potent antioxidant, it mitigates UV‐induced photodamage and inhibits melanin synthesis while promoting collagen biosynthesis to enhance cellular renewal and modulate inflammatory responses [4]. Therefore, it is widely utilized as an adjunctive therapeutic agent in the clinical management of melasma, photoaging, and anti‐aging interventions [5, 6]. This multifunctional profile has led to its frequent incorporation into skincare formulations, establishing it as a staple component in daily regimens for the general population.

Yellow light is a form of visible light with wavelengths ranging from 570 to 595 nm, capable of penetrating the skin to a depth of approximately 0.5 to 2 mm. It primarily targets keratinocytes, melanocytes, and endothelial cells within the epidermis and dermis, inducing photobiomodulation effects that can mitigate skin inflammation [7], inhibit the expression of matrix metalloproteinases [8], and decrease melanin production [9]. This modality is predominantly employed in the treatment of allergic dermatitis, seborrheic dermatitis, post‐inflammatory erythema [10] and melasma [9].

The treatment of melasma remains challenging due to variable efficacy, prolonged duration, and high recurrence rates among diverse therapeutic modalities. This has prompted many patients to seek safer, more effective, and convenient solutions for spot lightening, making vitamin C‐based skincare products and phototherapy their common choices. This study aims to compare the efficacy and safety profiles of monotherapy yellow light phototherapy versus its combination with 20% vitamin C in melasma treatment.

## 2 Material and Methods

### 2.1 Study Design and Participants

This study was a single‐center, randomized, double‐blind, placebo‐controlled clinical trial. A block randomization method was adopted, and the random allocation sequence was generated by statistical software. Research assistants screened eligible participants strictly according to the inclusion and exclusion criteria. After written informed consent was obtained, participant allocation was performed by an unblinded statistician. Allocation information was sealed in opaque envelopes. The investigational preparations were identical in appearance and labeled only with unique randomization codes. All study personnel and participants remained blinded throughout the trial, with no access to group allocation information, except for the unblinded statistician, to minimize allocation bias. To prevent accidental unblinding caused by skin irritation induced by vitamin C, participants received gradually escalating concentrations of vitamin C (2.5%, 5%, 10%) in the first 3 weeks, followed by a 20% concentration. Vitamin C powder and serum were individually packaged and freshly prepared immediately before use to reduce the incidence of skin irritation. Furthermore, all efficacy evaluations and follow‐up visits were conducted prior to product application. Data regarding subjective skin sensations, therapeutic effects, and adverse reactions after treatment were kept confidential from outcome assessors to further avoid the risk of unblinding. The protocol was approved by the Hospital Ethics Committee (in compliance with the Declaration of Helsinki, 2008 edition).

The primary outcome of this study was the change in the modified Melasma Area and Severity Index (mMASI) score from baseline to the end of the 3‐month intervention period [11]. Secondary outcomes included investigator‐assessed clinical improvement (Physician's Global Assessment, PGA) [12], subject‐assessed clinical improvement (Efficacy Satisfaction Assessment, SA), changes in pore size and skin texture evaluated by VISIA, as well as alterations in skin physiological parameters.

This study was conducted from August 2023 to August 2024. Participants were outpatients recruited from our hospital. We enrolled female patients aged 18–60 years with Fitzpatrick skin Types III–IV, bilateral symmetrical facial melasma, and good general health. All participants followed consistent sun protection guidance and completed all scheduled follow‐up visits over the 3‐month trial, though standardized routine monitoring of sunscreen and sun avoidance adherence was not implemented.

Exclusion criteria were as follows: pregnancy or lactation; known hypersensitivity to any ingredients of topical preparations; active dermatoses or cutaneous lesions at the treatment site; application of depigmenting agents within 14 days before baseline; cosmetic interventions including retinoid treatment, chemical peeling, and laser therapy administered in the preceding 3 months; enrollment in other clinical trials recently; continuous use of systemic hormonal contraceptives for no less than 3 months; impaired hepatic or renal function; concomitant systemic disorders interfering with skin metabolism; unreasonable therapeutic expectations; and refractory melasma, defined as less than 20% clinical improvement after a minimum of 3 months of first‐line treatment.

### 2.2 Interventions

All participants were randomized into two treatment groups: Group A (vitamin C combined with yellow light phototherapy) and Group B (vitamin C excipient combined with yellow light therapy). The study had a 12‐week observation period, with disease assessments conducted every 4 weeks. All subjects visited the hospital every 3–4 days for yellow light phototherapy (Wavelength 590 ± 10 nm, duration 10 min, power density 25 W/cm^2^, Comfort Expert, Chongqing Peninsula Medical Technology Co. Ltd., Chongqing, China). Following each session, a full‐face application of vitamin C essence or its vehicle (GALÉNIC COSMETICS LABORATORY 79 SAS, Toulouse, France) was administered. During the study, all participants used non‐whitening and non‐spot‐lightening facial creams and sunscreens uniformly provided by the research team, along with strict physical sun protection measures. Throughout the trial, the use of skin whitening agents, tranexamic acid, chemical peels, laser/radiofrequency, and any other medications or physical therapies that could affect the study outcomes was prohibited. All participants were required to take strict sun protection to avoid UV‐related bias. They applied unified sunscreen every 2–3 h outdoors and wore standard sun masks, with sun avoidance also needed beside indoor windows.

### 2.3 Research Evaluation

#### 2.3.1 Investigator Evaluation

At baseline (Week 0) and Week 12, two independent investigators, who were not involved in subject randomization or treatment allocation, assessed melasma severity using the mMASI score. Additionally, the PGA score was performed at Week 12. The total mMASI score was calculated as follows: mMASI = 0.3A(f)D(f) + 0.3A(lm)D(lm) + 0.3A(rm)D(rm) + 0.1A(c)D(c), with a possible range of 0 to 24. The abbreviations denote: f, forehead; lm, L. malar; rm, R. malar; c, chin. The scoring criteria were:

$$\begin{array}{c} A\;\left(\text{Area of involvement}\right):0=\text{absent},1=<10\%,2=10\%–29\%,\\ \;3=30\%–49\%,4=50\%–69\%,5=70\%–89\%,6=90\%–100\%. \end{array}$$

$$\begin{array}{c} D\;\left(\text{Darkness}\right):0=\text{absent},1=\text{slight},2=\text{mild},3=\text{marked},\\ \;4=\text{severe}. \end{array}$$

The PGA was scored on a 7‐point scale: 0 = clear; 1 = almost clear; 2 = marked improvement; 3 = moderate improvement; 4 = slight improvement; 5 = no improvement; 6 = worse.

#### 2.3.2 Participants Self‐Evaluation

Subjects completed the efficacy satisfaction assessment (SA) at Week 12. Patients' self‐evaluation defined as follows: 1 = very satisfied (improvement > 75%); 2 = Satisfied (50%–75% improvement); 3 = average (25%–49% improvement); 4 = not satisfied (improvement < 2 5%).

#### 2.3.3 Skin Detection

**FIGURE 1:**
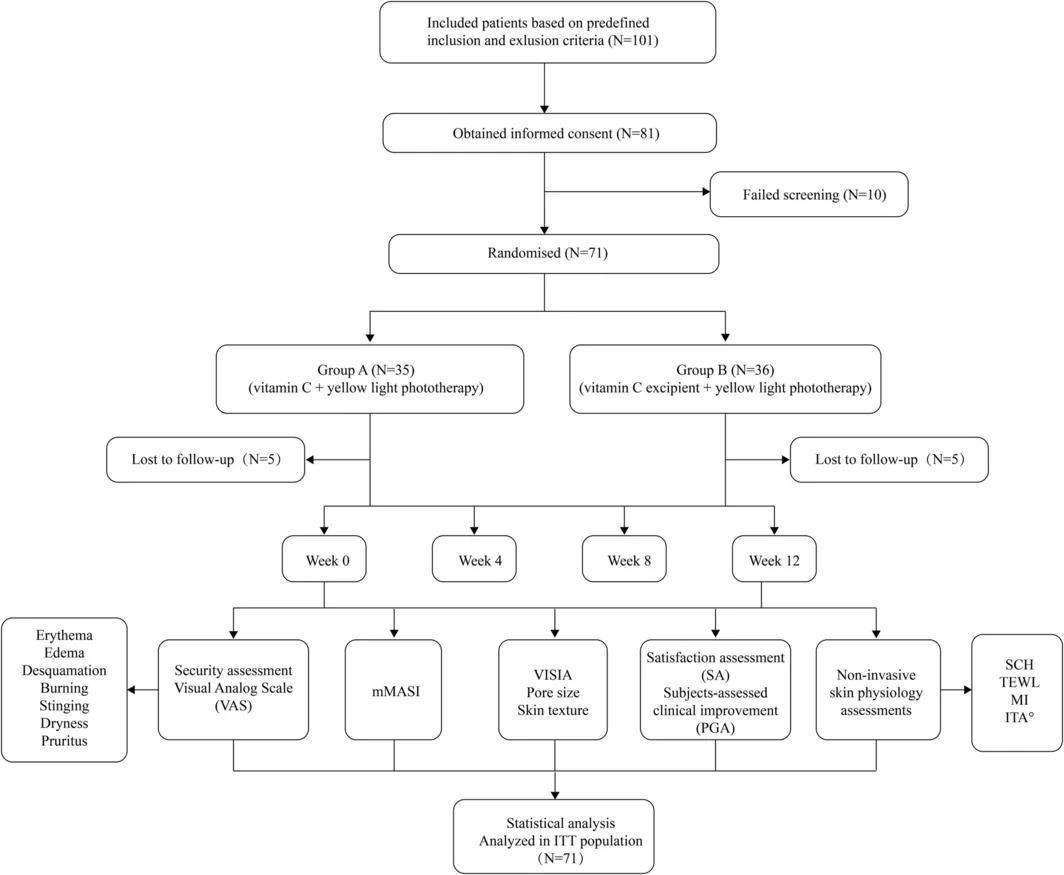
The workflow of our study. ITT, intention‐to‐treat analysis.

Standardized digital images were captured using a digital camera. Wood's lamp was employed to differentiate melasma subtypes. VISIA analysis (Canfield Imaging Systems, Canfield Scientific Inc., Parsippany Troy Hills, United States) and noninvasive skin assessments (TewameterTMHex, ColorimeterCM825, and Mexameter, Courage+Khazaka electronic GmbH, Cologne, Germany) were conducted at weeks 0, 4, 8, and 12. Stratum Corneum Hydration (SCH) denotes stratum corneum hydration, with higher values indicating greater water content. Transepidermal Water Loss (TEWL) stands for transepidermal water loss; higher values suggest impaired skin barrier function. Individual Typology Angle (ITA°) reflects skin tone, and larger values correspond to lighter skin color. Melanin Index (MI) represents cutaneous melanin level, where lower values indicate lighter skin tone. The workflow of our study is shown in Figure 1.

Prior to the VISIA examination, subjects are required to remove all facial makeup, cleanse their skin with plain water, and allow sufficient time for surface moisture to evaporate before proceeding with the assessment. This includes areas of erythema, hyperpigmentation, pores, and overall skin texture.

Prior to the initiation of noninvasive skin testing, subjects were acclimatized in a controlled environment maintained at a temperature range of 22°C–24°C and relative humidity between 40%–60% for a duration of 15–30 min. The parameters assessed during the testing included SCH, TEWL, MI, and ITA° values. To minimize measurement variability, all noninvasive skin assessments were conducted by the same individual.

#### 2.3.4 Security Assessment

To conduct safety assessments, the following parameters were recorded: skin adverse reactions following yellow light phototherapy and vitamin C application, adverse event (AE) reporting, and reasons for study withdrawal from baseline to Week 12. Not all changes constitute adverse events; only clinically significant changes related to yellow light therapy and vitamin C were defined as related adverse reactions.

Criteria for evaluating adverse reactions included investigator scores for erythema, edema, and desquamation, as well as subject scores for burning, stinging, dryness, and pruritus. Each assessment utilized the Visual Analog Scale (VAS), with scores ranging from 0 to 4 points, corresponding to no effect, mild effect, moderate effect, and severe effect, respectively.

### 2.4 Statistical Analysis

The primary outcome was the change in modified Melasma Area and Severity Index (mMASI) scores from baseline to the end of the 3‐month intervention. Based on an assumed effect size of 0.7, a statistical power of 80%, a two‐sided alpha level of 0.05, and an anticipated dropout rate of 8%, a total of 71 participants were recruited in this study. For continuous variables, the *t*‐test was applied if the normality assumption was satisfied; otherwise, the Mann–Whitney *U* test was used. Nominal categorical variables were analyzed using the chi‐squared test, with Fisher's exact test used when the chi‐square test's assumptions were violated. Repeatedly measured continuous variables will be analyzed using a mixed‐effects model for repeated measures (MMRM), by which missing data were imputed. The model includes change from baseline as the dependent variable, treatment group as the primary independent variable, and baseline value, visit, and visit‐by‐group interaction as covariates. Least squares means (LSM) and 95% confidence intervals (CI) will be estimated for each dose group at each visit. Treatment group differences versus control (LSM differences, 95% CI, and *p*‐values) will be calculated.

## 3 Results

### 3.1 Patient Characteristics

A total of 71 subjects were enrolled, with 35 in Group A and 36 in Group B. A total of 10 participants withdrew equally from Group A and Group B. One withdrew for emergent surgery, three missed follow‐ups due to work, and six failed to adhere to strict sun protection requirements. Demographic data, as shown in Table 1, indicated that all subjects were female, with a mean age of 45.9 ± 7.76 years and a mean disease duration of 3.87 ± 1.23 years. Regarding Fitzpatrick skin phototype, 42 subjects (59.2%) had Type III skin, and 29 subjects (40.8%) had Type IV skin. Based on Wood's lamp examination, melasma was classified as epidermal type in 19 subjects (26.8%), dermal type in 16 subjects (22.5%), and mixed type in 36 subjects (50.7%). Thirty‐seven subjects (52.1%) had concurrent telangiectasia, 28 subjects (39.4%) had a family history of melasma, and 57 subjects (80.3%) reported anxiety related to their condition. Comparisons between the two groups revealed no significant differences in age, disease duration, skin type, melasma subtype, or severity of anxiety (*p* > 0.5). The baseline mMASI scores were 8.77 ± 4.33 for Group A and 10.7 ± 6.05 for Group B at Week 0 (*p* > 0.5), as shown in Table 2. These data indicate that the baseline characteristics of the two groups were consistent, making them suitable for subsequent research and statistical analysis.

**TABLE 1:** Baseline demographics and disease characteristics.

	Group A (*N* = 35)	Group B (*N* = 36)	ALL (*N* = 71)	*p*
Age	46.1 (7.77)	45.7 (7.86)	45.9 (7.76)	0.833
Course of disease	3.94 (1.19)	3.81 (1.28)	3.87 (1.23)	0.712
Fitzpatrick				
III	18 (51.4%)	24 (66.7%)	42 (59.2%)	0.287
IV	17 (48.6%)	12 (33.3%)	29 (40.8%)	
Types of melasma				
Epidermal	9 (25.7%)	10 (27.8%)	19 (26.8%)	0.476
Mixed	16 (45.7%)	20 (55.6%)	36 (50.7%)	
Dermal	10 (28.6%)	6 (16.7%)	16 (22.5%)	
Family history				
N	19 (54.3%)	24 (66.7%)	43 (60.6%)	0.410
Y	16 (45.7%)	12 (33.3%)	28 (39.4%)	
Angiotelectasis				
N	18 (51.4%)	16 (44.4%)	34 (47.9%)	0.725
Y	17 (48.6%)	20 (55.6%)	37 (52.1%)	
Severity of anxiety				
No	6 (17.1%)	8 (22.2%)	14 (19.7%)	0.266
Mild	23 (65.7%)	19 (52.8%)	42 (59.2%)	
Moderate	5 (14.3%)	7 (19.4%)	12 (16.9%)	
Severe	1 (2.86%)	2 (5.56%)	3 (4.23%)	

*Note:* Group A defined as vitamin C combined with yellow light phototherapy. Group B defined as vitamin C excipient combined with yellow light therapy.

**TABLE 2:** Comparison of the main therapeutic effect evaluation indicators mMASI and PGA between the two groups.

	Group A (*N* = 35)	Group B (*N* = 36)	*p*
mMASI Week 0	8.77 (4.33)	10.7 (6.05)	0.132
mMASI Week 12	6.51 (3.67)	9.91 (5.80)	0.008
mMASI change^a^	−1.95 (2.11)	−0.69 (2.19)	0.027
PGA	4.48 (0.80)	5.06 (0.54)	0.002

*Note:* Group A defined as vitamin C combined with yellow light phototherapy. Group B defined as vitamin C excipient combined with yellow light therapy.

^a^ The variation of mMASI compared to the baseline.

### 3.2 Melasma Severity Score

Table 2 presents the results. At Week 12, the mMASI score of Group A (yellow light phototherapy combined with 20% vitamin C) was significantly lower than that of Group B (yellow light phototherapy combined with vehicle) (6.51 ± 3.67 vs. 9.91 ± 5.80, *p* = 0.008). Additionally, the reduction in mMASI score from baseline in Group A (1.95 ± 2.11) was significantly greater than that in Group B (0.69 ± 2.19, *p* = 0.027). These findings indicate that yellow light phototherapy combined with vehicle can reduce the mMASI score of melasma, while the combination of 20% vitamin C and yellow light phototherapy resulted in a more pronounced reduction. Thus, 20% vitamin C combined with yellow light therapy demonstrates superior efficacy to control group in the treatment of melasma.

### 3.3 Investigator‐Assessed Clinical Improvement

At the end of the 12‐week study, two independent investigators conducted the PGA to evaluate residual pigmentation. Table 2 showed that the PGA score of Group A (4.48 ± 0.80) was significantly lower than that of Group B (5.06 ± 0.54), with a statistically significant difference (*p* = 0.002). This indicates that the efficacy of 20% vitamin C combined with yellow light therapy was superior to that of the vehicle combined with yellow light therapy.

Additionally, Bland–Altman plot agreement testing was performed to assess the consistency between the mMASI scores (as shown in Figure S1) and PGA scores of the two investigators (as shown in Figure S2). The results revealed that all data points in the scatter plot fell within the 95% limits of agreement, confirming good inter‐rater consistency between the two investigators.

### 3.4 Subjects‐Assessed Clinical Improvement

At Week 12, patient satisfaction with treatment efficacy was assessed via questionnaire and statistically analyzed. As shown in Table 3, 42.86% of participants in Group A reported overall satisfaction versus 19.44% in Group B, while 22.85% in Group A and 8.34% in Group B indicated general satisfaction. Collectively, the total satisfaction rate was 65.71% in Group A and 27.78% in Group B, with 77.22% of participants in Group B expressing dissatisfaction. Significant intergroup differences in treatment satisfaction were observed (*p* = 0.003). These findings demonstrated that patients receiving 20% vitamin C combined with yellow light therapy achieved higher treatment satisfaction than those treated with vehicle plus yellow light therapy.

**TABLE 3:** Comparison of the subjects' satisfaction with the therapeutic effect after treatment.

	Group A (*N* = 35)	Group B (*N* = 36)	ALL (*N* = 71)	*p*
Degree of satisfaction				
Dissatisfaction	12 (34.29%)	26 (72.22%)	38 (53.52%)	0.003
General satisfaction	15 (42.86%)	7 (19.44%)	22 (30.98%)	
Satisfaction	8 (22.85%)	3 (8.34%)	11 (15.50%)	

*Note:* Group A defined as vitamin C combined with yellow light phototherapy. Group B defined as vitamin C excipient combined with yellow light therapy.

### 3.5 Skin Physiological Indicators

The results showed that there were no significant intergroup differences in SCH and TEWL at weeks 0, 4, 8, and 12 (*p* > 0.05), indicating that yellow light therapy combined with or without 20% vitamin C did not damage the skin barrier.

In terms of changes in MI from baseline, no significant difference was observed between Group A and Group B at Week 4 (*p* > 0.05). At Weeks 8 and 12, the adjusted mean differences versus baseline were −11.02 (95% CI: −15.30, −6.74) versus −1.13 (95% CI: −5.41, 3.16), and −13.07 (95% CI: −17.82, −8.32) versus −3.16 (95% CI: −7.86, 1.54), respectively, with significant intergroup differences (*p* < 0.05) (Table 4). These findings suggested that significant between‐group differences in MI of melasma lesions emerged since Week 8, with a greater reduction in Group A, and the difference was most prominent at Week 12.

**TABLE 4:** Comparison of skin physiological indices between the two groups.

	Group A (*N* = 35)	Group A (*N* = 36)
**SCH**		
W4		
LSM^a^ (95% CI)	−1.01 (−3.54, 1.51)	−2.10 (−4.65, 0.45)
LSM difference (95% CI)	1.09 (−2.51, 4.68)	
*p*	0.547	
W8		
LSM (95% CI)	−1.63 (−4.65, 1.39)	−3.15 (−6.16, −0.14)
LSM difference (95% CI)	1.52 (−2.75, 5.79)	
*p*	0.478	
W12		
LSM (95% CI)	−0.67 (−3.68, 2.34)	−4.02 (−7.00, −1.05)
LSM difference (95% CI)	3.35 (−0.88, 7.58)	
*p*	0.119	
**TEWL**		
W4		
LSM (95% CI)	0.70 (−0.07, 1.47)	0.62 (−0.15, 1.40)
LSM difference (95% CI)	0.08 (−1.02, 1.18)	
*p*	0.886	
W8		
LSM (95% CI)	1.75 (0.89, 2.61)	0.90 (0.04, 1.76)
LSM difference (95% CI)	0.85 (−0.37, 2.07)	
*p*	0.169	
W12		
LSM (95% CI)	1.64 (0.87, 2.40)	1.67 (0.92, 2.42)
LSM difference (95% CI)	−0.04 (−1.11, 1.04)	
*p*	0.948	
**MI**		
W4		
LSM (95% CI)	−6.31 (−9.92, −2.71)	−1.87 (−5.53, 1.78)
LSM difference (95% CI)	−4.44 (−9.63, 0.75)	
*p*	0.092	
W8		
LSM (95% CI)	−11.02 (−15.30, −6.74)	−1.13 (−5.41, 3.16)
LSM difference (95% CI)	−9.89 (−16.02, −3.77)	
*p*	0.002	
W12		
LSM (95% CI)	−13.07 (−17.82, −8.32)	−3.16 (−7.86, 1.54)
LSM difference (95% CI)	−9.91 (−16.65, −3.16)	
*p*	0.005	
**ITA**		
W4		
LSM (95% CI)	0.93 (0.07, 1.78)	0.84 (−0.02, 1.71)
LSM difference (95% CI)	0.09 (−1.13, 1.31)	
*p*	0.886	
W8		
LSM (95% CI)	3.11 (1.74, 4.49)	0.44 (−0.93, 1.80)
LSM difference (95% CI)	2.68 (0.74, 4.61)	
*p*	0.007	
W12		
LSM (95% CI)	6.23 (4.38, 8.08)	1.23 (−0.60, 3.05)
LSM difference (95% CI)	5.01 (2.40, 7.61)	
*p*	< 0.001	

*Note:* Group A defined as vitamin C combined with yellow light phototherapy. Group B defined as vitamin C excipient combined with yellow light therapy.

^a^ Least squares means.

For changes in ITA° relative to baseline, no significant difference was found at Week 4 (*p* > 0.05). Significant intergroup differences were detected at weeks 8 and 12, with corresponding mean differences of 3.11 (95% CI: 1.74, 4.49) versus 0.44 (95% CI: −0.93, 1.80) and 6.23 (95% CI: 4.38, 8.08) versus 1.23 (95% CI: −0.60, 3.05) (*p* < 0.05) (Table 4). Collectively, skin tone differed significantly between the two groups starting from Week 8, with a more obvious lightening effect in Group A, and the maximal difference was presented at Week 12.

### 3.6 Pore Size and Skin Texture Assessed by VISIA

Finally, the effects of yellow light therapy and/or vitamin C on skin photoaging were evaluated by assessing changes in pores and skin texture obtained via VISIA analysis.

As shown in Table 5, the least squares means in pores from baseline were 5.70 (95% CI: 4.38, 7.03) versus 1.51 (95% CI: 0.18, 2.83) at Week 8, and 9.53 (95% CI: 7.73, 11.33) versus 2.38 (95% CI: 0.61, 4.16) at Week 12, with statistically significant intergroup differences at both time points (*p* < 0.001). These results indicated that both groups showed improvement in pores at weeks 8 and 12, with a greater degree of improvement observed in Group A compared to Group B.

**TABLE 5:** Comparison of pore and texture detected by VISIA between the two groups.

	Group A (*N* = 35)	Group B (*N* = 36)
**Pore**		
W4		
LSM (95% CI)^a^	1.77 (0.578, 2.96)	1.50 (0.29, 2.71)
LSM difference (95% CI)	0.27 (−1.43, 1.97)	
*p*	0.751	
W8		
LSM (95% CI)	5.70 (4.38, 7.03)	1.51 (0.18, 2.83)
LSM difference (95% CI)	4.19 (2.32, 6.07)	
*p*	< 0.001	
W12		
LSM (95% CI)	9.53 (7.73, 11.33)	2.38 (0.61, 4.16)
LSM difference (95% CI)	7.15 (4.62, 9.67)	
*p*	< 0.001	
**Texture**		
W4		
LSM (95% CI)	1.28 (0.07, 2.48)	0.86 (−0.36, 2.08)
LSM difference (95% CI)	0.42 (−1.30, 2.13)	
*p*	0.629	
W8		
LSM (95% CI)	4.02 (2.09, 5.96)	1.53 (−0.39, 3.46)
LSM difference (95% CI)	2.49 (−0.24, 5.22)	
*p*	0.073	
W12		
LSM (95% CI)	7.68 (5.63, 9.74)	3.29 (1.27, 5.31)
LSM difference (95% CI)	4.39 (1.51, 7.27)	
*p*	0.004	

*Note:* Group A defined as vitamin C combined with yellow light phototherapy. Group B defined as vitamin C excipient combined with yellow light therapy.

^a^ Least squares means.

In addition, statistical analysis of changes in skin texture from baseline revealed a significant difference between the two groups at Week 12, with least squares means of 7.68 (95% CI: 5.63, 9.74) for Group A and 3.29 (95% CI: 1.27, 5.31) for Group B (*p* = 0.004). This finding demonstrated that skin texture improved in both groups at Week 12, and the improvement was more pronounced in Group A.

**FIGURE 2:**
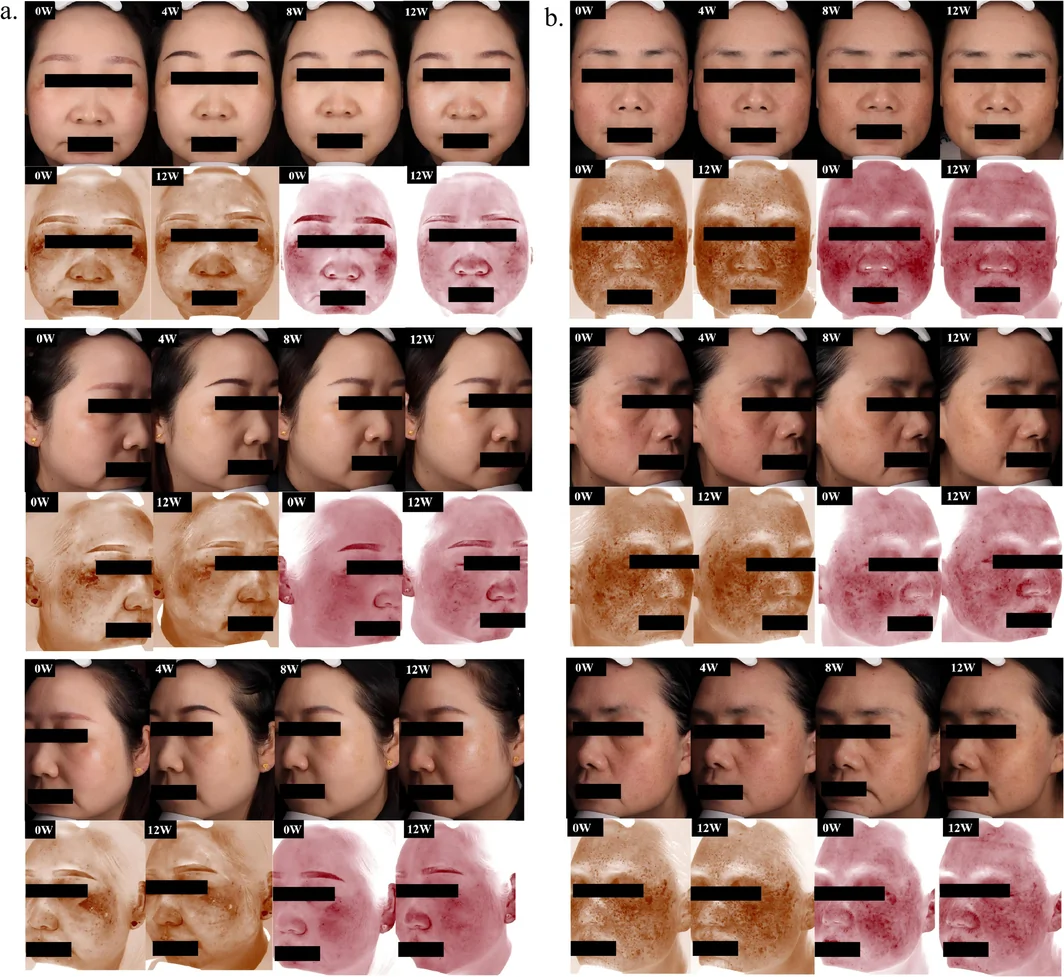
(a) VISIA images from a representative subject in Group A following 12 weeks of combined 20% vitamin C and yellow light phototherapy exhibited lightening of facial brown patches, with significant improvement in both pigmented spots and red areas. (b) VISIA images from a representative subject in Group B after 12 weeks of treatment revealed only slight changes in facial brown patches, pigmented spots, and red areas.

Figure 2 showed representative participants from Group A and Group B. The VISIA images present the clinical changes under standard lighting, as well as the specific alterations in brown spots and red areas.

### 3.7 Safety Assessment

In Group A, 6 subjects experienced immediate erythema and stinging following the first application of 20% vitamin C, resulting in an adverse reaction incidence rate of 17.14% (6/35). Among these, 5 cases (14.28%, 5/35) presented with mild stinging and erythema, which completely resolved within 1 day after application of a moisturizer. One case (2.86%, 1/35) developed mild erythema and stinging immediately after the second application, which subsided rapidly after moisturizer application. Additionally, 1 case (2.86%, 1/35) exhibited moderate stinging and erythema immediately post‐first treatment; this was managed with oral loratadine 10 mg once daily, facial mask compress, and moisturization, leading to complete recovery after 7 days without residual post‐inflammatory erythema or pigmentation. No adverse reactions were observed in the control group.

## 4 Discussion

Current treatment options for melasma include oral tranexamic acid, topical hydroquinone, and the Kligman's formula, which are considered effective pharmacological choices. However, these therapies carry risks such as increased thrombotic potential, reduced menstrual flow [13], and skin irritation [14]. Laser therapy is recommended for stable melasma but still poses a risk of post‐inflammatory hyperpigmentation (PIH). These limitations underscore the urgent need for novel therapies that are both effective and safe. The combination of 20% vitamin C and yellow light phototherapy provides a valuable adjunct to existing treatment regimens, addressing the substantial unmet need for safer and more effective melasma management.

It is well‐established that excessive UV exposure plays a critical role in the pathogenesis of melasma [2]. Melasma often coexists with photoaging or is itself considered one of the cutaneous manifestations of photoaging [15]. Thus, beyond retinoids, vitamin C stands out as the most evidence‐based agent with dual efficacy in skin brightening/depigmentation and anti‐photoaging [5]. It can inhibit UV‐induced melanin production and stimulate collagen neogenesis, and has been extensively studied and utilized in the treatment of melasma revious studies have used vitamin C concentrations ranging from 3.75% to 30%, with the optimal concentration being formulation‐dependent [5, 16, 17, 18]. In most cases, a vitamin C concentration exceeding 8% is required to elicit a measurable biological effect in the skin. Given its intradermal half‐life of approximately 4 days, an application frequency of once every 3 days ensures that a substantial portion of the vitamin C remains unmetabolized, thereby sustaining its therapeutic activity [19]. Studies have shown that 20% is the optimal concentration for maximizing the percutaneous absorption of vitamin C. Concentrations exceeding this threshold do not enhance its biological efficacy but may, conversely, lead to adverse effects such as skin irritation [19]. To date, only a few studies have evaluated the efficacy and safety of 20% vitamin C [19, 20]. This study utilized a 20% vitamin C essence, with subjects instructed to apply it every 2–3 days. The mMASI and PGA results tentatively validated the reliability and efficacy of this approach. In vitro studies have shown that 20% represents the maximum concentration for optimal transdermal absorption of vitamin C [19, 21]. Another notable characteristic of vitamin C in skincare products is its extreme instability, making it prone to oxidative degradation. This study employed a packaging design where the solution and vitamin C powder were separately packaged and mixed immediately before use, minimizing oxidative degradation of vitamin C.

Low‐level light therapy (LLLT) is based on the widespread photobiomodulatory effects of light‐emitting diode (LED) light, which enhance mitochondrial adenosine triphosphate (ATP) production, cellular signaling, and growth factor synthesis, while reducing oxidative stress. Currently, LLLT has been extensively studied in fields such as skin rejuvenation, acne, androgenetic alopecia, wound healing, and body contouring [22]. Dai et al. demonstrated that 590 nm LED irradiation improves erythema and pigmentation in melasma by inhibiting angiogenesis in human microvascular endothelial cells [9]. Mpofana et al. reported that sequential application of 633 nm and 830 nm LED light irradiation alleviates melasma in individuals with Fitzpatrick skin Types V and VI [23].

To date, the efficacy of vitamin C monotherapy and LED phototherapy for melasma has been individually validated in clinical trials [23, 24, 25, 26]. Combination of topical treatments with other modalities may enhance outcomes. Previous studies have shown that combining vitamin C with iontophoresis [27], ultrasound [28] or microneedling [16] reduces the MASI and improves clinical efficacy, primarily leveraging the enhanced penetration and absorption of vitamin C into the skin facilitated by these physical therapies. In contrast, our study aimed to combine 20% vitamin C with LED yellow light irradiation for a distinct purpose. As is well‐known, melasma often coexists with compromised skin barrier function [29]. Our protocol involved applying a 20% vitamin C essence, followed by 10‐min irradiation with 590 ± 10 nm yellow light 10 min later. We hypothesized that low‐intensity LED yellow light would exert biostimulatory effects on the skin, inducing cellular functional changes to regulate and ameliorate inflammation in the epidermis and superficial dermis, thereby achieving anti‐inflammatory, depigmenting, and skin barrier‐repairing effects. However, the exact mechanism of action remains unexplored in this study. Our results showed that both vehicle plus yellow light and vitamin C plus yellow light achieved reductions in mMASI scores; the decrease was statistically greater in the vitamin C combination group (1.95 ± 2.11 vs. 0.69 ± 2.19). Despite this statistically significant intergroup difference, the absolute between‐group gap in pigment improvement was modest, suggesting the 20% vitamin C combination regimen yields mild additional pigment‐lightening benefits relative to vehicle plus yellow light for melasma management. Additionally, secondary assessments of patient satisfaction further confirmed this conclusion. Although yellow light alone contributes to depigmentation and skin brightening, the combination with vitamin C exhibited more significant effects, as evidenced by statistical analyses of skin physiological parameters (MI and ITA°).

Our study also focused on changes in skin texture and pore appearance. Results showed that both groups had improved pores from baseline, with a significantly greater reduction in the vitamin C plus yellow light group than in the vehicle plus yellow light group (9.53 vs. 2.38; 95% CI: 7.73–11.33 and 0.61–4.16, respectively; *p* < 0.001). Additionally, both groups showed improved skin texture from baseline, with a significantly greater improvement in the vitamin C plus yellow light group than in the vehicle plus yellow light group (7.68 vs. 3.29; 95% CI: 5.63–9.74 and 1.27–5.31, respectively; *p* = 0.004). These findings are consistent with those of Fitzpatrick et al., who conducted a 12‐week study involving 10 melasma subjects treated topically with 10% vitamin C. Their results revealed a significant reduction in photoaging scores in the vitamin C group compared to the placebo group, indicating that vitamin C can improve cutaneous photoaging [30].

Additionally, this study also investigated the impact of 20% vitamin C combined with yellow light phototherapy on the skin barrier. TEWL was selected as a key parameter for barrier assessment. The results showed no statistically significant differences in TEWL between the vitamin C combined with yellow light group and the yellow light alone group at weeks 0, 4, 8, and 12. This indicates that neither yellow light therapy nor 20% vitamin C treatment impaired the skin barrier function, consistent with the findings of most previous studies [19, 20, 25].

Finally, we also evaluated the safety and tolerability of vitamin C and phototherapy. It is well‐known that vitamin C‐containing skincare products, particularly those with high‐concentration vitamin C, are more irritating to the skin, requiring careful use to allow the skin to gradually develop tolerance. In this study, to minimize skin irritation caused by vitamin C, we adjusted the concentration from 2.5% to 10% during the initial application. After the skin had developed tolerance, we then used the standard 20% concentration. The results showed an adverse reaction incidence rate of 17.14% (6/35) for erythema and stinging. Hwang et al. used a formulation containing 25% L‐ascorbic acid and chemical penetration enhancers to treat melasma, enrolling 40 subjects. Among these, 30 developed stinging, 23 reported burning, and 6 experienced pruritus, with an adverse reaction incidence rate significantly higher than that in our study [31]. This discrepancy is speculated to be related to the higher concentration of vitamin C used.

In summary, our study reveals that the combination of 20% vitamin C and yellow light phototherapy produced statistically significant improvements in melasma compared with yellow light monotherapy, though the absolute reduction in mMASI was relatively small. This combined regimen did not impair skin barrier function, and participants generally tolerated the treatment well, with no severe adverse events recorded.

## 5 Conclusions

In this single‐center, randomized, double‐blind study, combined 20% vitamin C plus yellow light achieved statistically better efficacy than yellow light monotherapy for melasma in female patients. Statistically significant improvements in mMASI, PGA scores, skin texture and pore appearance were observed, suggesting the combination exerts depigmenting effects alongside mild anti‐aging benefits, with acceptable safety. While intergroup differences reached statistical significance, the absolute mMASI improvement was limited. Collectively, this combination regimen may offer certain clinical value for melasma management.

## 6 Limitation

This single‐center trial carries multiple limitations. Restricted to one clinical site, its findings lack broad generalizability. The maximum 12‐week follow‐up also prevents evaluation of long‐term therapeutic efficacy. In addition, the relatively small sample size requires larger cohorts to corroborate the present observations. Furthermore, without standardized monitoring of sun‐protection compliance, unmeasured ultraviolet exposure may confound treatment‐related melasma outcomes.

## Author Contributions

Conceptualization: D.D., X.J. Data curation: D.D., X.L. Formal analysis: Y.H., S.W. Investigation: Y.H., S.W. Methodology: X.L., D.D., X.L. Resources: X.J. Software: D.D. Supervision: X.J. Visualization: D.D., X.L. Writing‐original draft: D.D. Writing‐review and editing: D.D., X.J.

## Funding

This study was financially supported by Galénic Cosmetics Laboratory SAS, Paris, France, as well as the Chengdu Science and Technology Bureau (2025‐YF09‐00010‐SN) and the 2024 Central Government Guided Local Science and Technology Development Project for Regional Innovation System Construction, Multifunctional Intelligent Photoelectric Energy Platform (2024ZYD0275).

## Ethics Statement

The study approved by the Ethics Committee of West China Hospital of Sichuan University.

## Consent

The photos of the participants involved in this article have all obtained their informed consent and they have signed the informed consent form.

## Conflicts of Interest

The commercial sponsor had no authority to influence study design, data collection, statistical analysis, manuscript preparation, or the decision to submit this article for publication. All raw research data are independently controlled and fully owned by the authors. The authors declare no conflicts of interest.

## Supporting information


**Figure S1:** Bland–Altman Plot for assessment of inter‐rater reliability in mMASI scores between two groups.
**Figure S2:** Bland–Altman Plot for assessment of inter‐rater reliability in PGA scores between two groups.

## Data Availability

The data that support the findings of this study are available from the corresponding author, upon reasonable request.
