# Supplementary material for: Efficacy and Safety of Yellow Light Monotherapy Versus Yellow Light Combined With 20% Vitamin C for the Treatment of Melasma: A Single‐Center, Randomized, Double‐Blind, Placebo‐Controlled Clinical Study
Source: J Cosmet Dermatol. 2026 Aug 23;25(9):e71146. doi: 10.1111/jocd.71146 (PMC13501208; doi:10.1111/jocd.71146)
Supplement: Supplementary file 1 [file JOCD-25-e71146-s001.docx]

Figure S1:Bland-Altman Plot for assessment of inter-rater reliability in mMASI scores between two groups.

Figure S2:Bland-Altman Plot for assessment of inter-rater reliability in PGA scores between two groups.
